# A Critically Ill Child with Gangrenous Appendicitis Masquerading as Hollow Viscous Perforation

**DOI:** 10.1155/2020/8857058

**Published:** 2020-12-26

**Authors:** Ashish Lal Shrestha, Santosh Maharjan, Anil Dev Pant, Pankaj Bahadur Nepali

**Affiliations:** ^1^Department of Pediatric Surgery, Grande International Hospital, Tokha Road, Kathmandu, Nepal; ^2^Department of Radiodiagnosis, Grande International Hospital, Tokha Road, Kathmandu, Nepal; ^3^Department of Pathology, Grande International Hospital, Tokha Road, Kathmandu, Nepal

## Abstract

**Background:**

Severe complications of acute appendicitis (AA) hitherto well described are less common in clinical practice nowadays. When a septic child is encountered with a short history of abdominal symptoms and disproportionate signs of peritonitis further complicated by radiological findings causing a diagnostic conundrum, management becomes exceedingly demanding. *Case Presentation*. A 10-year-old previously healthy boy presented to the emergency room with generalized abdominal pain associated with fever and jaundice for a day. Blood workup revealed leucopenia, hyperbilirubinemia, hyponatremia, and elevated CRP. Initial radiological evaluation suggested hollow viscous perforation. He was diagnosed to have hollow viscous perforation peritonitis in severe sepsis. At laparotomy, generalized peritoneal contamination was found, the source of which could be traced down to the gangrenous perforated appendix.

**Conclusion:**

Complicated appendicitis, in children, can present with baffling findings. Timely identification of an ill child, adequate workup, prompt resuscitation, and source control are imperative for a successful outcome.

## 1. Introduction

Appendicitis can present with complications like gangrene, perforation, and peritonitis with the incidence of perforation being reported ~30% in children [1]. While the risks are more for younger children due to inexpressibility and language coherence, amongst the older ones, the delayed presentation seems to be accountable [1].

The incidence of adult appendicular perforation presenting with pneumoperitoneum is estimated to be 0-7% while no similar data seems to exist in children [2].

In 70% of adults with peritonitis, pneumoperitoneum is an expected finding that can be linked with perforated peptic ulcer in most, perforated sigmoid diverticulitis in a few, and an anastomotic leak in postoperative patients in the rest, thereby tending to overlook an appendicular etiology [2, 3].

Confounded by limitations in history taking and nonspecific examination findings, it becomes even a bigger diagnostic challenge in children. We present an interesting case of a boy presenting with generalized peritonitis in severe sepsis with pneumoperitoneum initially thought to have peptic ulcer perforation and on further evaluation found to have a gangrenous perforated appendicitis.

## 2. Case Report

A 10-year-old Nepalese boy presented to the emergency room (ER) for sudden periumbilical pain that became generalized over the day associated with fever, bilious vomiting, abdominal distension, and obstipation. General examination revealed fever (101 degrees F), tachycardia (120 beats per minute), and tachypnea (40 breaths per minute). His blood pressure was 90/60 mmHg, and capillary refill time was 3 seconds. He looked dehydrated with a dry and coated tongue and seemed to be in notable distress. Abdominal examination revealed generalized distension with tenderness, guarding, and absent bowel sounds on auscultation.

His hemogram showed leucopenia (total leukocyte count (TLC): 2550 cells/Cu.mm and differential leukocyte count: polymorphs: 58%, lymphocytes: 14%, eosinophils: 1%, monocytes: 26%, and basophils: 1%) and elevated C-reactive protein (CRP): 90 mg/l.

Biochemical tests showed moderate hyponatremia (Na+: 128 mmol/l) and normal liver panel except for mild hyperbilirubinemia (2.6 mg/dl). Urinalysis was normal.

**Figure 1:**
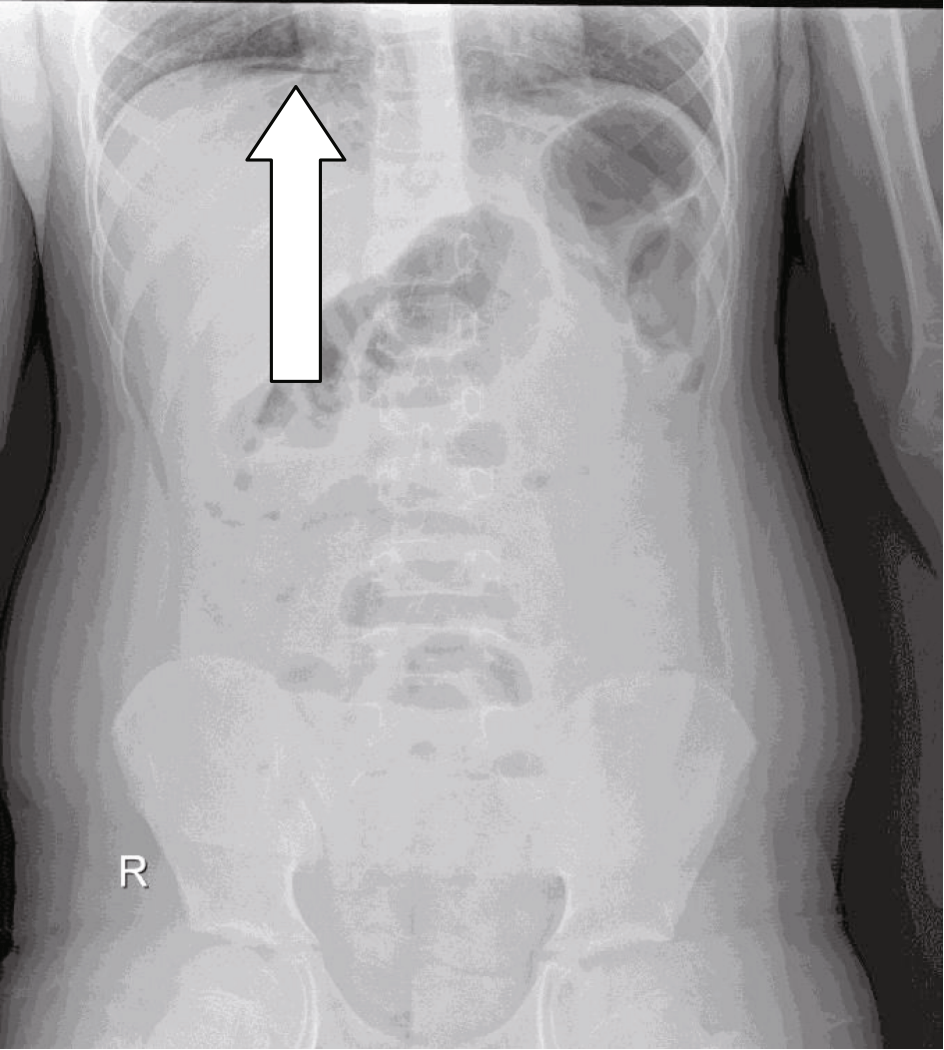
Plain abdominal erect radiograph showing subdiaphragmatic free air.

Abdominal radiograph revealed free air under the diaphragm as shown in Figure 1. Ultrasound abdomen showed distended gassy bowel.

He was diagnosed to have hollow viscous perforation peritonitis with a source probably in the proximal gastrointestinal tract. Nasogastric decompression was initiated along with aggressive fluid resuscitation, first dose intravenous broad-spectrum antibiotics (piperacillin-tazobactam, amikacin, and metronidazole), and analgesics.

**Figure 2:**
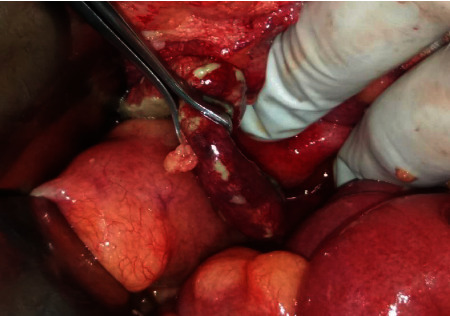
Intraoperative appearance of a gangrenous perforated appendix.

He underwent an emergency exploratory midline laparotomy under general anesthesia. Intraoperatively generalized peritonitis was found with putrid pus and fibrinous flakes in the subdiaphragmatic regions, Morrison's pouch, both paracolic gutters, and pelvis. The appendix was found to be gangrenous and perforated at its base as shown in Figure 2. Appendectomy and thorough peritoneal irrigation with normal saline was performed. Although the base was perforated, the adjoining caecum was healthy along with a portion of appendicular stump that allowed transfixing sutures done doubly with 3-0 Vicryl. A 20Fr ADK drain was left to drain the pelvis.

The fascia was repaired with interrupted 1 Vicryl without tension and skin with staplers.

Postoperatively, he was monitored in PICU (Pediatric Intensive Care Unit) with continuation of supportive care. While in PICU, his vital signs remained stable with the maintenance of intake-output balance and correction of metabolic acidosis.

On postoperative day (POD) 3, his TLC improved to 7210 cells/Cu.mm, CRP remained >90 mg/l, total bilirubin was 2.2 mg/dl, and serum sodium levels normalized (Na+:136 m mol/l).

On postoperative day 5, TLC was 6650 cells/Cu.mm, CRP was 23 mg/l, and total bilirubin was 0.9 mg/dl. Peritoneal pus had grown Pseudomonas aeruginosa that was sensitive to the antibiotics that he was receiving. Since the drain output was insignificant, it was removed.

He had an uneventful postoperative course, and after a total of 7 days, intravenous antibiotics were discontinued and he was subsequently discharged.

**Figure 3:**
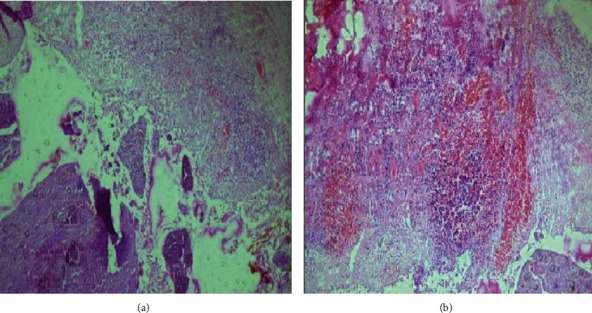
Histopathological appearance of the appendix at 40x magnifications showing (a) fecalith and mucosal ulceration and (b) full-thickness inflammation and myonecrosis.

The histopathology showed appendicular mucosa with suppuration, ulceration, and full-thickness inflammation along with myonecrosis. The lumen contained fecal material. The findings were suggestive of gangrenous appendicitis with periappendiceal inflammation as shown in Figure 3.

**Figure 4:**
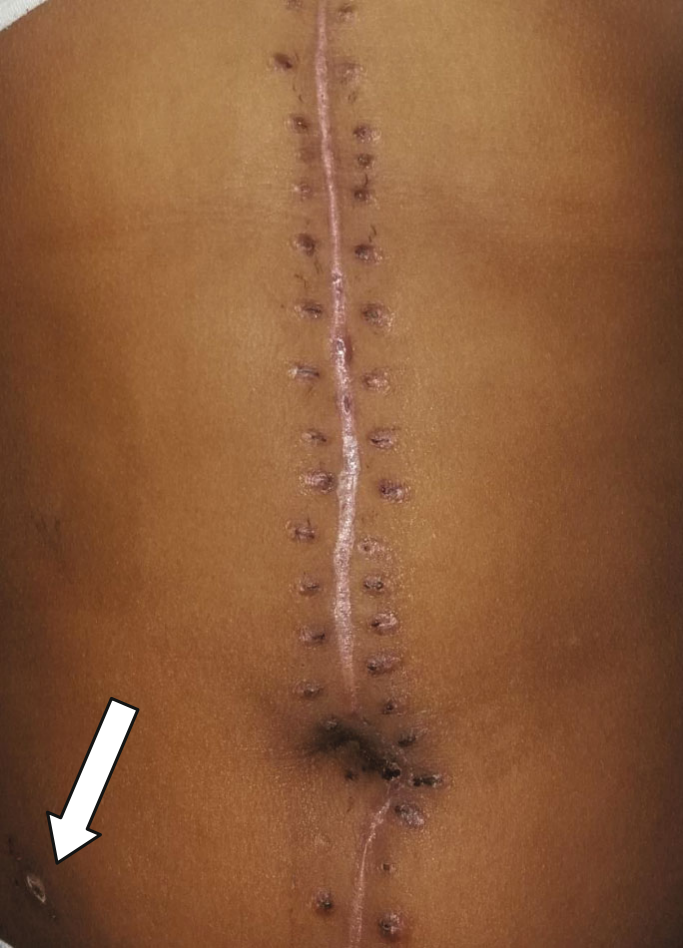
Midline laparotomy scar with drain insertion site, after surgical clip removal at 1-week follow-up.

At follow-up a week later, he had recovered well. At the same visit, skin staplers were removed as shown in Figure 4.

## 3. Discussion

Peritonitis in children is a well-established entity, and appendicular perforation as a cause is also widely known [4, 5]. The temporal profile in the natural history of appendicitis results in various stages of inflammation including complications like gangrene and perforation, the prediction of which is not easy [1].

Generally, the intraluminal lymphoid hyperplasia is the pathogenic mechanism causing AA. But any process that could occlude its lumen causing an increase in luminal pressure could induce inflammation. This would allow bacterial translocation, transmural suppuration, and eventual necrosis with perforation [2]. However, pneumoperitoneum remains an uncommon finding with appendicular perforation owing to the lumen that is already obstructed [2].

In few patients, the intestinal free air may overcome the obstruction, escape into the peritoneal cavity, and manifest as free air in plain abdominal erect films. In others, the bacteria involved in periappendicular abscess could be responsible for gas formation [2]. Though minimal (3 ml) air is sufficient to manifest pneumoperitoneum on a plain and erect abdominal radiograph, the finding coexisting with a perforated appendix is rather rare even in adult patients [6]. The fact that no large studies are reported in children could mean that it is even more exceedingly rare in children [7].

Whatever the cause of pneumoperitoneum or peritonitis, the initial workup and resuscitation always take precedence over the diagnostic confirmation. When presented with a critically ill child with such findings, the first and foremost step is to attain hemodynamic stability, with a prompt and adequate resuscitation meanwhile continuing further evaluation to plan a definite management [8]. Pain control with appropriate analgesics and first dose antibiotics need not be delayed till the definitive diagnosis is made.

If a plain radiograph at ER suggests pneumoperitoneum with background findings of peritonitis in sepsis, the next definitive step is source control. As with our patient, we decided to proceed with a midline laparotomy and complete exploration in order to clear pus from the deeper peritoneal recesses and to avoid abscesses in the postoperative period apart from trying to avoid missing an upper gastrointestinal pathology. A formal exploration in this case was unavoidable in view of (1) unknown focus of infection and free air and (2) general peritoneal contamination.

## 4. Conclusion

Appendicular perforation peritonitis presenting with sepsis and free peritoneal air on a plain radiograph is a rare phenomenon or rather an uncommon presentation of a much common disease process. The finding may present with dilemma in diagnosis and surgical approach. However, with generalized peritoneal contamination, complete abdominal exploration is imperative to avoid missing findings and to attain adequate source control. Regardless of surgical findings, the initial steps of resuscitation and workup are paramount.

## Data Availability

Data used to support the findings of this study are available from the corresponding author upon request.

## Abbreviations

AA: Acute appendicitis
PICU: Pediatric intensive care unit
TLC: Total leukocyte count
CRP: C-reactive protein.

## References

[1] Howell E. C., Dubina E. D., Lee S. L. (2018). Perforation risk in pediatric appendicitis: assessment and management. *Pediatric Health, Medicine and Therapeutics*.

[2] Wong C. W. Y., Chung P. H. Y., Lan L. C. L., Wong K. K. Y. (2015). Acute appendicitis presenting as pneumoperitoneum in a teenage boy undergoing chemotherapy: Figure 1. *BML Case Reports*.

[3] ŞSahin S., ÇCavusolu T., Kubat M., ÇCalisş H. (2016). A rare cause of pneumoperitoneum: perforated appendicitis. *Journal of Ayub Medical College Abbottabad*.

[4] Mutabazi E., Bonane A., Ndibanje A. J., Rickard J. (2018). Epidemiological study of peritonitis among children and factors predicting mortality at a tertiary referral hospital in Rwanda. *East and Central African Journal of Surgery*.

[5] Osifo O. D., Ogiemwonyi S. O. (2011). Peritonitis in children: our experience in Benin City, Nigeria. *Surgical Infections*.

[6] Chan K. K. (1962). Acute appendicitis with pneumoperitoneum. *Singapore Medical Journal*.

[7] Duman L. (2014). Pneumoperitoneum. *Annals of Pediatric Surgery*.

[8] Tan B., Wong J. J.-M., Sultana R. (2019). Global case-fatality rates in pediatric severe sepsis and septic shock: a systematic review and meta-analysis. *JAMA Pediatrics*.

